# ﻿Three new species of *Psectrocladius* Kieffer (Diptera, Chironomidae) from China

**DOI:** 10.3897/zookeys.1239.145869

**Published:** 2025-05-20

**Authors:** Wenbin Liu, Yaning Tang, Jiaxin Nie, Ziming Shao, Wei Cao, Chuncai Yan

**Affiliations:** 1 Tianjin Key Laboratory of Conservation and Utilization of Animal Diversity, Tianjin Normal University, Tianjin, 300387, China Tianjin Normal University Tianjin China

**Keywords:** China, Chironomidae, identification key, new species, taxonomy

## Abstract

Three new species within the genus *Psectrocladius* Kieffer, 1906, namely Psectrocladius (Mesopsectrocladius) wangi Liu, **sp. nov.**, Psectrocladius (Psectrocladius) gracilis Liu, **sp. nov.**, and Psectrocladius (Psectrocladius) malum Liu, **sp. nov.**, are described and illustrated based on morphological characteristics of adult male specimens. Furthermore, a comprehensive taxonomic key for the identification of adult males of all known *Psectrocladius* species recorded in China is provided.

## ﻿Introduction

Kieffer (1906) erected the genus *Psectrocladius* with *Orthocladiuspsilopterus* Kieffer, 1906 as the type species. *Psectrocladius* occurs in a wide variety of aquatic biotopes, predominantly in standing or slow-flowing waters (Cranston et al. 1989). Adults of the genus *Psectrocladius* can be distinguished from other Orthocladiinae by their combinations of bare eyes, well-developed pulvilli, and straight Cu_1_ (Cranston et al. 1989). The pupae of this genus are unique due to their long posterior spines on tergites V and VIII, the absence of frontal setae and pedes spurii B, and their differentiation from other Orthocladiinae with a thoracic horn, well-developed fringe, and at least one lamelliform (or strong) L seta (Coffman et al. 1986). In addition, *Psectrocladius* are relatively uniform and distinctive in their larval stage, characterized notably by the palmate structure of S1, which is unique, as well as by the combination of a large ventromental plate, a simple premandible, and spurs on the procercus (Andersen et al. 2013).

Through comprehensive morphological studies of larval-adult associations, Wülker (1956) established a taxonomic framework for the genus *Psectrocladius*, proposing its division into three distinct subgenera: *Psectrocladius* s. str. Kieffer, *Monopsectrocladius* Wülker, and *Allopsectrocladius* Wülker. This revision was accompanied by a diagnostic key for the identification of male adults. Subsequently, Roback (1957) expanded our understanding of the genus through detailed ontogenetic studies, describing three previously unrecognized species. These taxonomic descriptions were particularly significant as they included comprehensive morphological characterizations of female imagines, pupal exuviae, and larval instars, thereby providing a complete life history perspective for these newly discovered taxa.

The taxonomic understanding of *Psectrocladius* has been progressively refined through a series of significant contributions. Sæther (1969) made notable advancements through his examination of Canadian specimens from Walden Lake, documenting three species, including one novel taxon. Laville (1971), following meticulous examination of type specimens of *Psectrocladiusbarbatipes* Kieffer, 1923, made a crucial taxonomic determination. His analysis revealed that the larval morphology of this species exhibited intermediate characteristics between the *P.psilopterus* and *P.dilatatus* species groups, which correspond to the subgenera *Psectrocladius* and *Allopsectrocladius*, respectively. This morphological intermediacy warranted the establishment of a new subgenus, *Mesopsectrocladius* Laville. In the United Kingdom, Pinder (1978) significantly contributed to the regional taxonomy by developing a comprehensive identification key for British *Psectrocladius* species, encompassing nine species distributed across the three established subgenera: *Psectrocladius*, *Monopsectrocladius*, and *Allopsectrocladius*. Langton (1980) further enhanced diagnostic capabilities by providing distinct subgeneric keys for both pupal and adult stages, along with specific keys for the subgenus Psectrocladius, covering eight species. This work was subsequently supplemented by Langton’s (1985) revisionary study of *Psectrocladiuslimbatellus* and associated specimens. Parallel research efforts in Russia and Japan have substantially expanded the known diversity of this genus. Russian researchers (Zelentsov 1980; Zelentzov and Makarchenko 1988; Makarchenko and Makarchenko 2003) documented four species, while Japanese investigations (Yamamoto 2004) significantly increased this number with records of fourteen species, demonstrating the genus’s extensive distribution and morphological diversity across the Palaearctic region.

The study of *Psectrocladius* in China has evolved through several significant contributions since the initial taxonomic work. Kieffer (1923) provided the first record of this genus in China with the description of *Psectrocladiusformosae* Kieffer, 1923 from Taiwan Province. Subsequent research by Wang and Zheng (1996) expanded our knowledge through the description of *Psectrocladiuslongipennis* Wang et Zheng, 1996, based on specimens collected from Qinghai Province. Wang (2000) further documented the genus’ diversity in China, reporting several unidentified taxa along with two adult specimens, one larval specimen, and two records requiring taxonomic verification. Recent investigations in Xizang Autonomous Region have yielded important records of immature stages, with Ge et al. (2024) documenting both larval and pupal specimens of *Psectrocladiusnevalis* Akhrorov, 1977.

Current taxonomic understanding recognizes seven *Psectrocladius* species in China, with complete adult descriptions available for only four taxa: *P.formosae* Kieffer, 1923, *P.longipennis* Wang et Zheng, 1996, *P.obvius* (Walker, 1856), and *P.sokolovae* Zelentzov et Makarchenko, 1988. The remaining species - *P.limbatellus* (Holmgren, 1869), *P.nevalis* Akhrorov, 1977, and *P.barbimanus* (Edwards, 1929) - are known from either immature stages or incomplete records (Ashe and O’Connor 2012; Wang et al. 2020; Ge et al. 2024). The present study significantly contributes to the Chinese *Psectrocladius* fauna through the description and illustration of three new species. Additionally, we provide a comprehensive diagnostic key for the identification of adult males of all known *Psectrocladius* species recorded in China, facilitating future taxonomic studies in this region.

## ﻿Material and methods

The morphology and terminology are based on Sæther (1980). The material examined was mounted on slides using the procedure outlined by Sæther (1969). When three or more specimens were measured, the measurements are provided as the range and mean, with the number of observed specimens in parentheses if it differs from the number (*N*) stated at the beginning of the description. Color descriptions pertain to ethanol-preserved specimens. The holotype of all new species is deposited at the College of Life Sciences, Tianjin Normal University, Tianjin, China (TJNU).

## ﻿Taxonomy

### ﻿ Psectrocladius (Mesopsectrocladius) wangi

Taxon classificationAnimaliaDipteraChironomidae

Liu sp. nov.

5EBC6A25-0D60-586C-90EF-41A99B49B30A

https://zoobank.org/D8057D4F-8A80-44BB-BF8A-7040EC8EBB2A

Figs 1 , 2 , 3

#### Material examined.

***Holotype***: • male (TJNU No. 04724), China, Fujian Province, Fujian Agriculture and Forestry University, 26°05'17"N, 119°18'43"E, 22.IV.1993, X.H. Wang, light trap. ***Paratype***: • two male, same data as holotype; one male, China, Guizhou Province, Fanjing Mountain protection temple, 27°55'N, 108°41'E, 28.V.2002, R.L. Zhang, light trap; • one male, China, Fujian Province, Shanghang County, Gutian Town. 25°13'28"N, 116°49'23"E, 4.V.1993. X. H. Wang, sweep.

#### Diagnosis.

The anal tergite is wider at the top and narrower at the bottom with a rounded posterior margin. The anal point is short and rounded at the tip, thumb-like. The femur setae are thick, resembling spines.

#### Description.

Male (*N* = 5, unless otherwise stated in brackets).

Total length 3.37–3.88, 3.56 mm. Wing length 1.78–2.10, 1.92 mm. Total length/wing length 1.75–1.96, 1.86. Wing length/length of profemur 2.08–2.28, 2.23. The thorax is yellow-brown with brown markings, scutellum and the posterior half of the scutellum are lighter in color. The abdomen is brown.

**Figure 1.**
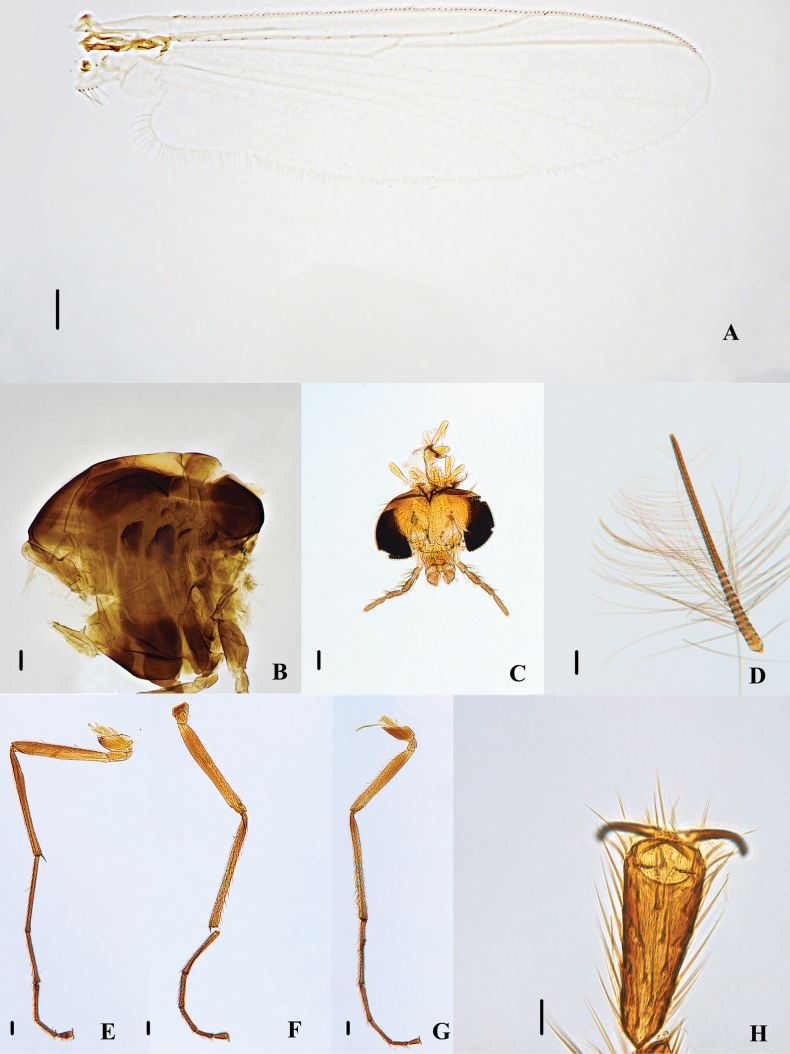
Psectrocladius (Mesopsectrocladius) wangi Liu, sp. nov., holotype male **A** wing **B** thorax **C** head **D** antenna **E** foreleg **F** midleg **G** hindleg **H** tarsus V. Scale bars: 100 μm (**A, B, C, D, E, F, G**); 20 μm (**H**).

Head (Fig. 1C). AR 1.66–1.78, 1.72. Temporal setae 14–17, 15; including 3–7, 6 inner verticals; 4–9, 6 outer verticals; and 6–10, 8(3) postorbitals. Clypeus with 21–37, 29 setae. Tentorium 139–172, 160 μm long. Palpomere lengths (II–V in μm): 51–75, 62; 81–117, 94; 78–107, 91; 121–151, 137; The ratio of the length of V to III: 1.29–1.62, 1.43.

Thorax (Figs 1B, 3B). Antepronotals with 5–12, 9 setae, acrostichals 5–7, 6, dorsocentrals 17–28, 23. prealars 4–8, 5. Scutellum with 6–12, 10 setae.

**Figure 2.**
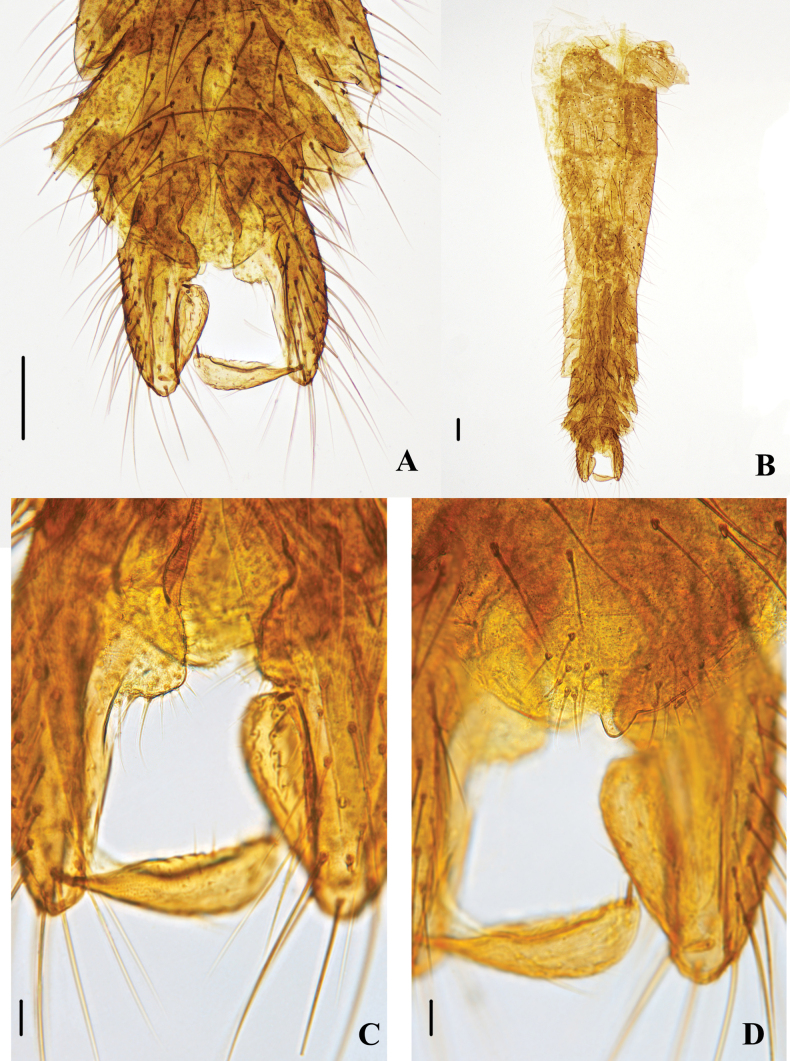
Psectrocladius (Mesopsectrocladius) wangi Liu, sp. nov., holotype male **A** hypopygium **B** abdomen **C** volsella **D** anal point. Scale bars: 100 μm (**A, B**); 20 μm (**C, D**).

**Figure 3.**
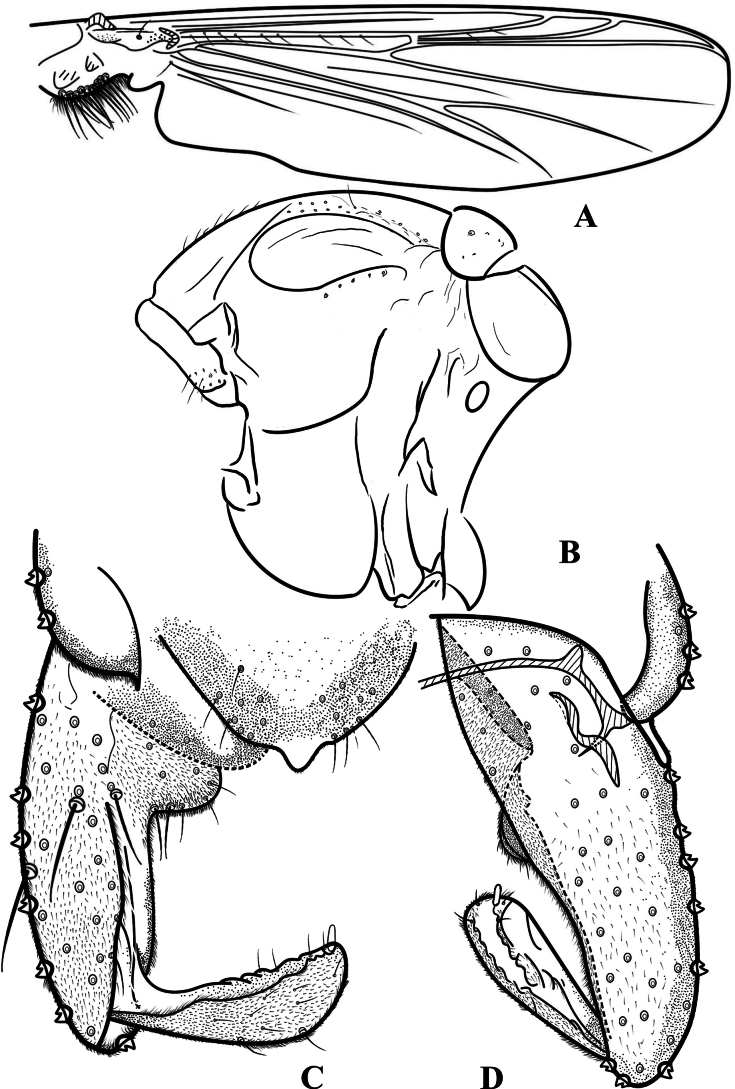
Psectrocladius (Mesopsectrocladius) wangi Liu, sp. nov., holotype male **A** wing **B** thorax **C** hypopygium, dorsal view **D** hypopygium, ventral view.

Wing (Figs 2A, 3A). Anal lobe developed. VR 1.17–1.21, 1.19. Costa extension 36–49, 43 µm. The end of R_2+3_ is between R_1_ and R_4+5_. Radius with 8–15, 11 setae. R_1_ with 2–6, 4 setae. Squama with 13–17, 16 setae. Brachiolum with one seta.

Legs (Fig. 1E–G). Tarsomeres without bristles, hind leg with long bristles. Front tibia with one spur, 75–85, 81 µm long. Mid tibial with 2 spurs, the long one 53–60, 58 µm and the other is one-third of it, 20–25, 23 µm. Hind tibia with one spur, 70–80, 73 µm long. The hind tibial comb with 10–14, 12 spurs. Tarsus I and II of mid leg with two pseudospurs. Tarsus III of mid legs with 0–1 pseudospurs. Tarsus I and II of hind leg with two pseudospurs. Tarsus III of hind legs with 0–1 pseudospurs. Lengths (in μm) and proportions of legs as in Table 1.

**Table 1.** Lengths (in μm) and proportions of legs of Psectrocladius (Mesopsectrocladius) wangi Liu, sp. nov., male (*N* = 5).

	**ti**	**fe**	**ta1**	**ta2**	**ta3**
P1	990–1188, 1074	822–927, 864	567–742, 625	339–432, 378	269–324, 288
P2	823–984, 882	762–889, 822	371–453, 398	242–270, 242	180–229, 197
P3	819–945, 878	681–817, 754	568–697, 604	373–445, 399	262–328, 283
	**ta5**	**ta4**	**LR**	**BV**	**SV**
P1	110–130, 118	136–171, 152	0.55–0.62, 0.58	2.70–2.76, 2.74	2.85–3.27, 3.12
P2	107–119, 113	112–130, 123	0.43–0.47, 0.45	2.94–3.16, 3.07	4.13–4.48, 4.29
P3	111–123, 115	158–192, 176	0.65–0.74, 0.69	2.26–2.35, 2.30	2.53–2.85, 2.71

Hypopygium (Figs 2A, C, D, 3C, D). Tergite IX with 14–18, 16 setae located in the lower part of anal tergite equably. Anal tergite wider at the top and narrower at the bottom, and margo inferior smooth. The end of anal point smooth and as wide as the base of anal point, thumb-like. Transverse sternapodeme 125–150, 129 µm long centrally slightly arched, with prominent oral projections. Virga 73–85, 75 µm long, club-shaped. The dorsal volsella is salient, the ventral volsella is arc-shaped and bears a row of setae. Gonocoxite 272–315, 287 µm long, Gonostylus 111–148, 126 µm long, outer margin with conspicuous setal fringe. Megaseta 15–30, 24 µm long. HR 2.13–2.51, 2.46; HV 2.62–3.19. 2.85.

#### Distribution.

China (Fujian, Guizhou).

#### Etymology.

Named after Xinhua Wang, in honor of his contributions to the study of Chironomidae, noun in nominative case.

#### Remarks.

The subgenus Psectrocladius (Mesopsectrocladius) currently comprises two recognized species globally (Ashe and O’Connor 2012): P. (M.) barbatipes Kieffer, 1923 and P. (M.) seiryuheius Sasa, Suzuki & Sasai, 1999. These taxa exhibit distinct morphological characteristics that facilitate their differentiation. Psectrocladius (M.) seiryuheius is particularly notable for the complete absence of both anal point and virga, representing a unique apomorphic condition within the subgenus.

Comparative morphological analysis reveals that P. (M.) barbatipes shares certain similarities with the newly described species. However, it can be distinguished by several key characteristics: (1) the presence of a less developed and partially reduced anal point, and (2) a significantly higher antennal ratio (AR > 2.0, following Laville’s 1971 description). Furthermore, P. (M.) barbatipes exhibits distinctive chaetotaxy on its mid legs, characterized by exceptionally long setae, as evidenced by its high bristle ratio (BR > 3.0). This morphological feature suggests an adaptation potentially related to specific ecological requirements or behavioral patterns.

### ﻿ Psectrocladius (Psectrocladius) gracilis

Taxon classificationAnimaliaDipteraChironomidae

Liu sp. nov.

D44E2125-085A-5BD2-8114-D456E05467BA

https://zoobank.org/1FFE46C2-ED89-4F81-959D-7F95D38E4FCC

Figs 4 , 5 , 6

#### Material examined.

***Holotype***: • male (TJNU No.1210), China, Inner Mongolia Autonomous Region, Ulansuhai Nur, 40°55'19"N, 108°50'66"E, IV.1982, X.H. Wang. Sweep. ***Paratypes***: • two males, same data as holotype.

#### Diagnosis.

Thorax and abdomen dark. Anal tergite inverted triangle, both sides with a reticulate pattern; anal point mid-length, uniform thickness, gonostylus narrow and long.

#### Description.

Male (*N* = 3, unless stated).

Total length 3.63–3.66, 3.65 mm. Wing length 2.00, 2.15 (2) mm. Total length/wing length 1.69, 1.82 (2). Wing length/length of profemur 2.43, 2.36 (2). Thorax dark brown to black, abdomen yellowish brown (Fig. 5B).

**Figure 4.**
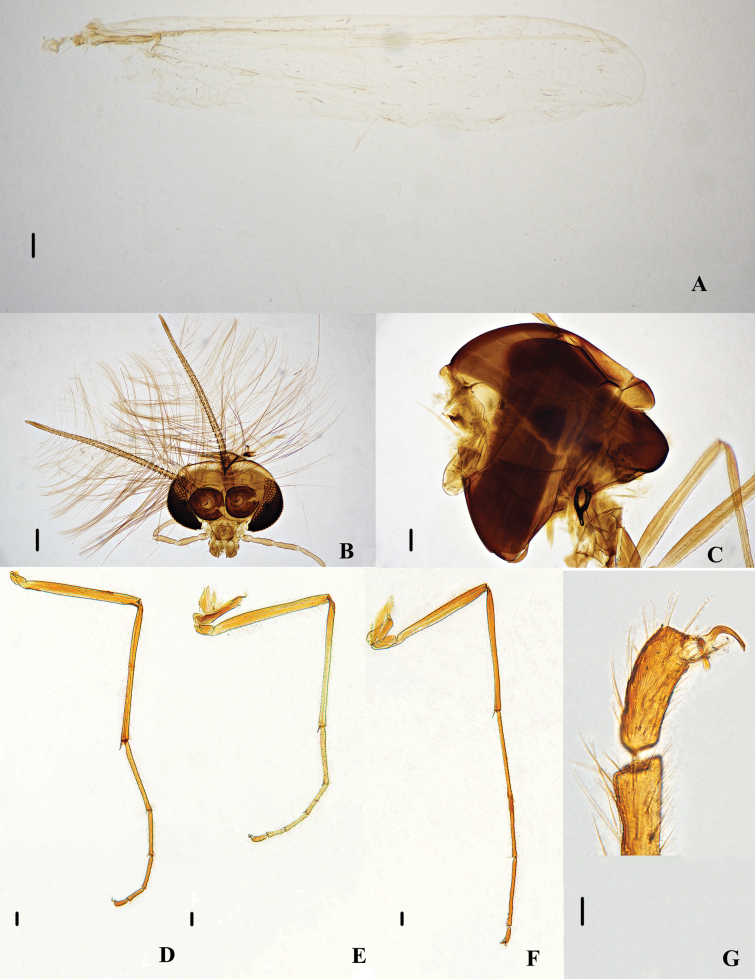
Psectrocladius (Psectrocladius) gracilis Liu, sp. nov., holotype male **A** wing **B** head **C** thorax **D** foreleg **E** midleg **F** hindleg **G** tarsus V. Scale bars: 100 μm (**A, B, C, D, E, F**); 20 μm (**G**).

Head (Fig. 4B). AR 1.67–1.97, 1.80. Temporal setae 10–12, 11; including 2–4, 3 inner verticals; 3–6, 4 outer verticals; and 2–5, 4 postorbitals. Clypeus with 9–11, 10 setae. Tentorium 150–164, 155 μm long. Palpomere lengths (II–V in μm): 48–62, 56; 86–107, 97; 110–130, 113; 131–160, 148. The ratio of the length of V to III: 1.35–1.86, 1.55.

Thorax (Figs 4C, 6B). Antepronotals 4–10, 7, acrostichals absent, dorsocentrals 10–16, 13, prealars 4–8, 6. Scutellum with 4–6, 5 setae.

Wing (Fig. 4A, 6A). Anal lobe developed. VR 1.16, 1.23 (2). Costa extension 55, 58 (2) µm. The end of R_2+3_ is between R_1_ and R_4+5_. Radius with 5 (1) setae. Squama with 29 (1) setae. Brachiolum with one seta.

Legs (Fig. 4E–G). Front tarsomeres without bristles, tibial comb with one spur, 68–83, 74 µm long, mid tibia with two spurs, one is 53–55, 53 µm long, one is thin and small, 8–10, 9 µm long. Hind tibia with one spur, 70–75, 73 µm long. The tibial comb of mid legs with 13–17, 14 spurs. Tarsus I of mid legs with two pseudospurs. Tarsus II of mid legs with 1–2 pseudospurs. Tarsus III of mid legs with two pseudospurs. Tarsus I and II of hind legs with two pseudospurs. Lengths (in μm) and proportions of legs as in Table 2.

**Table 2.** Lengths (in μm) and proportions of legs of Psectrocladius (Psectrocladius) gracilis Liu, sp. nov., male (*N* = 3).

	**Fe**	**ti**	**ta1**	**ta2**	**ta3**
P1	823–910, 880	949–1102, 1031	549–575, 560	333–351, 341	240–269, 256
P2	703–858, 776	757–865, 827	381–408, 392	227–240, 210	171–176, 172
P3	683–792, 752	816–913, 875	628–678, 652	408–440, 428	289–315, 300
	**ta4**	**ta5**	**LR**	**BV**	**SV**
P1	134–150, 141	77–124, 105	0.52–0.58, 0.55	1.67–2.08, 1.87	3.23–3.50, 3.41
P2	108–125, 115	96–99, 97	0.45–0.50, 0.48	3.04–3.47, 3.23	3.83–4.44, 4.08
P3	181–205, 191	103–126, 115	0.71–0.77, 0.75	2.15–29, 2.20	2.39–2.63, 2.50

**Figure 5.**
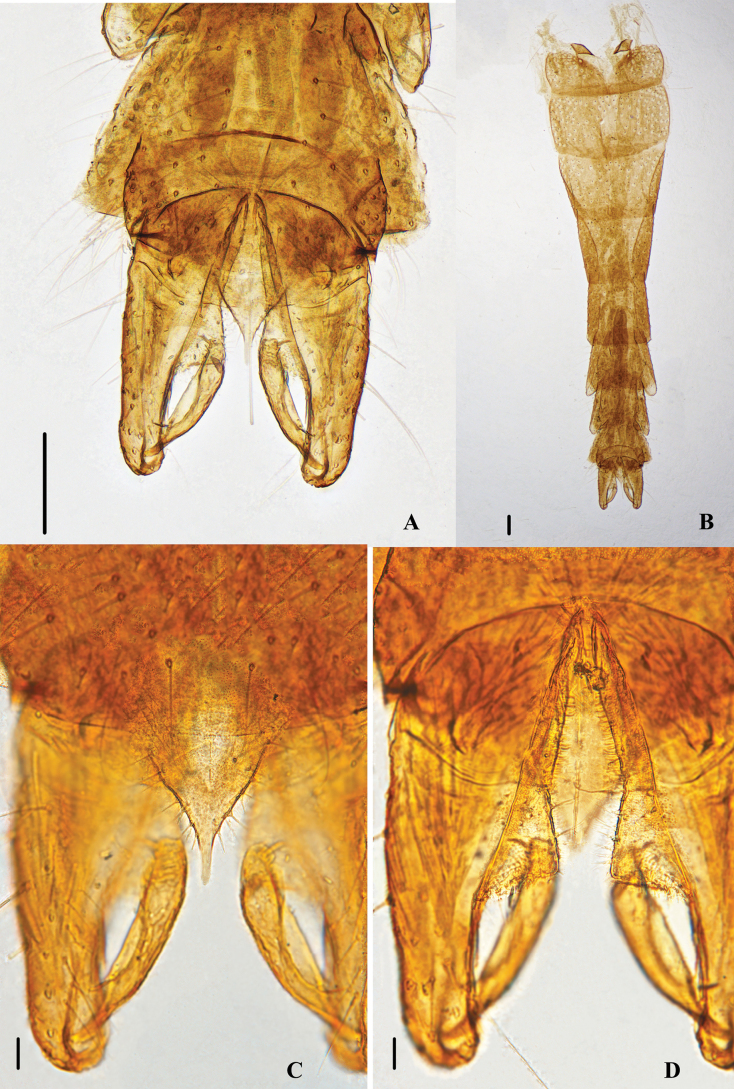
Psectrocladius (Psectrocladius) gracilis Liu, sp. nov., holotype male **A** hypopygium **B** abdomen **C** anal point **D** volsella. Scale bars: 100 μm (**A, B**); 20 μm (**C, D**).

**Figure 6.**
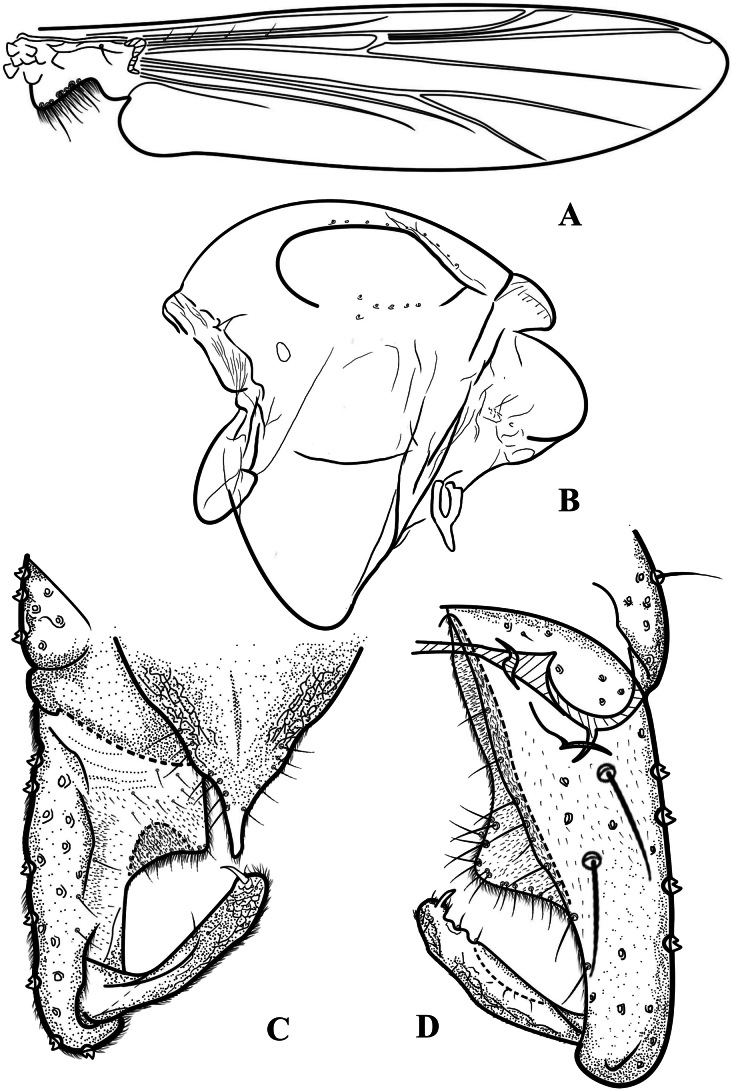
Psectrocladius (Psectrocladius) gracilis Liu, sp. nov., holotype male **A** wing **B** thorax **C** hypopygium dorsal view **D** hypopygium ventral view.

Hypopygium (Figs 5A, C, D, 6C, D). Tergite IX with 13–19, 15 setae ascending along both sides of the base of anal point. Tergite paratergital 10–15, 12 setae. Anal tergite inverted triangle, both sides with a reticulate pattern. Anal point mid-length, 20–29, 25 µm long, uniform thickness. Transverse sternapodeme 115–138, 124 µm long, central slightly arched, with well-developed bilateral ossified processes. Virga 75–80, 78 µm, the top of virga a small part is hook-shaped. Dorsal volsella square-shaped, ventral circular. Both sides of volsella with setae. Gonocoxite 250–276, 266 µm. Gonostylus 117–130, 123 µm, megaseta 12–17, 15 µm. HR 2.08–2.26, 2.16; HV 2.79–3.11, 2.97.

#### Distribution.

China (Inner Mongolia).

#### Etymology.

From the Latin, *gracilis*, narrow and thin, referring to the character of tergite IX and gonostylus, adjective in the nominative singular.

#### Remark.

The newly described species, Psectrocladius (Psectrocladius) gracilis Liu, sp. nov., exhibits significant morphological affinities with P. (P.) jintuoctadecimus (Sasa, 1996) in several diagnostic characters. These shared characteristics include: (1) the structural configuration of the inferior volsella, (2) the distinctive reticulate patterning on the lateral aspects of tergite XI, and (3) comparable measurements of both antennal ratio (AR) and leg ratio (LR). However, P. (P.) gracilis Liu, sp. nov. can be readily distinguished by its elongate and slender anal point and gonostylus, which contrast markedly with the nearly triangular morphology observed in P. (P.) jintuoctadecimus (Sasa, 1996), particularly in the latter’s characteristic broad-tipped gonostylus that significantly exceeds its basal width.

Furthermore, while P. (P.) gracilis Liu, sp. nov. shares certain anal point characteristics with P. (P.) limbatellus (Holmgren, 1869), the two species are clearly differentiated by their gonostylus morphology. The gonostylus of P. (P.) gracilis Liu, sp. nov. is diagnostically characterized by its notably narrow and attenuated structure, representing a distinct morphological divergence from the gonostylus configuration observed in P. (P.) limbatellus.

### ﻿ Psectrocladius (Psectrocladius) malum

Taxon classificationAnimaliaDipteraChironomidae

Liu sp. nov.

6BEB00D7-86BE-53A0-BA01-0A05B7D4542E

https://zoobank.org/07A8C464-9E51-408C-BE83-7764045EBBA4

Figs 7 , 8 , 9

#### Material examined.

***Holotype***: • male (TJNU No.12521), China, Inner Mongolia Autonomous Region, Xilinhot City, 43°57'29"N, 116°03'41"E, 1020 m, 10.VIII.1997, H.H. Li, sweep.

#### Diagnosis.

The base of gonocoxite with a small convex, the mid-upper part of gonocoxite with obvious concave, the outlines of both the left and right sides create a shape reminiscent of an apple. The end of inferior volsella pendulous and tongue-shaped.

#### Description.

Male (*N* = 1).

Total length 4.07 mm. Wing length 2.04 mm. Total length/wing length 2.00. Wing length/length of profemur 2.32. Thorax yellow to brown, with dark brown patterns. Abdomen II–V yellow, abdomen VI–VIII yellowish brown.

**Figure 7.**
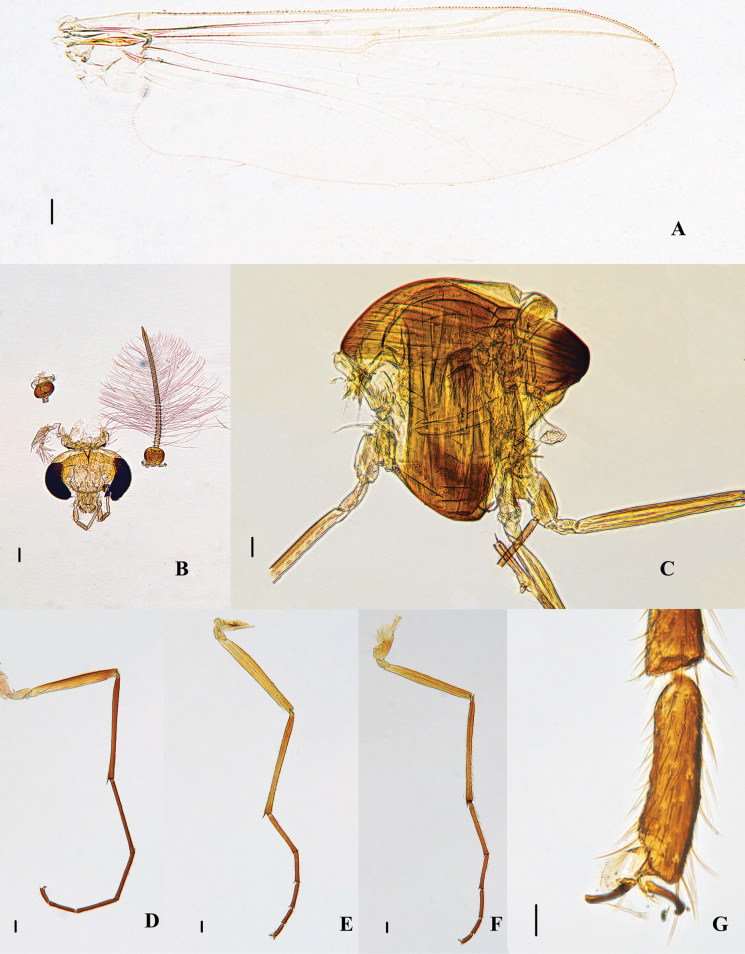
Psectrocladius (Psectrocladius) malum Liu, sp. nov., holotype male **A** wing **B** head **C** thorax **D** foreleg **E** midleg **F** hindleg **G** tarsus V. Scale bars: 100 μm (**A, B, C, D, E, F**); 20 μm (**G**).

Head (Fig. 7B). AR 1.70. Temporal setae 13; including 4 inner verticals; 3 outer verticals; and 6 postorbitals. Clypeus with 16 setae. Tentorium 116 μm long. Palpomere lengths (II–V in μm): 54, 102, 89, 203. The ratio of the length of V to III: 2.00.

Thorax (Figs 7C, 9B). Antepronotum with 7 setae, acrostichals absent, dorsocentrals 6, prealars 6. Scutellum two setae.

Wing (Figs 7A, 9A). Anal lobe developed. VR 1.17. Costa extension 43 µm. The end of R_2+3_ is between R_1_ and R_4+5_. Radius with 9 setae. R_1_ with one seta. Squama with two setae. Brachiolum with two setae.

Legs (Fig. 7E–G). Front legs, mid legs and hind legs each with one tibial spur, length is 70, 55, 65 µm. The tibial comb of hind legs with 17 spurs. Tarsus I of fore legs with one pseudospurs. Tarsus I, II and III of mid legs with two pseudospurs. Tarsus I, II and III of hind legs with two pseudospurs. Lengths (in μm) and proportions of legs as in Table 3.

**Table 3.** Lengths (µm) and proportion of legs of Psectrocladius (Psectrocladius) malum Liu, sp. nov., male (*N* = 1).

	fe	ti	ta1	ta2	ta3	ta4	ta5	LR	BV	SV
P1	873	1060	551	330	268	164	122	0.52	2.81	3.51
P2	800	880	387	243	211	144	128	0.44	2.85	4.34
P3	832	973	702	440	373	246	146	0.72	2.45	2.57

**Figure 8.**
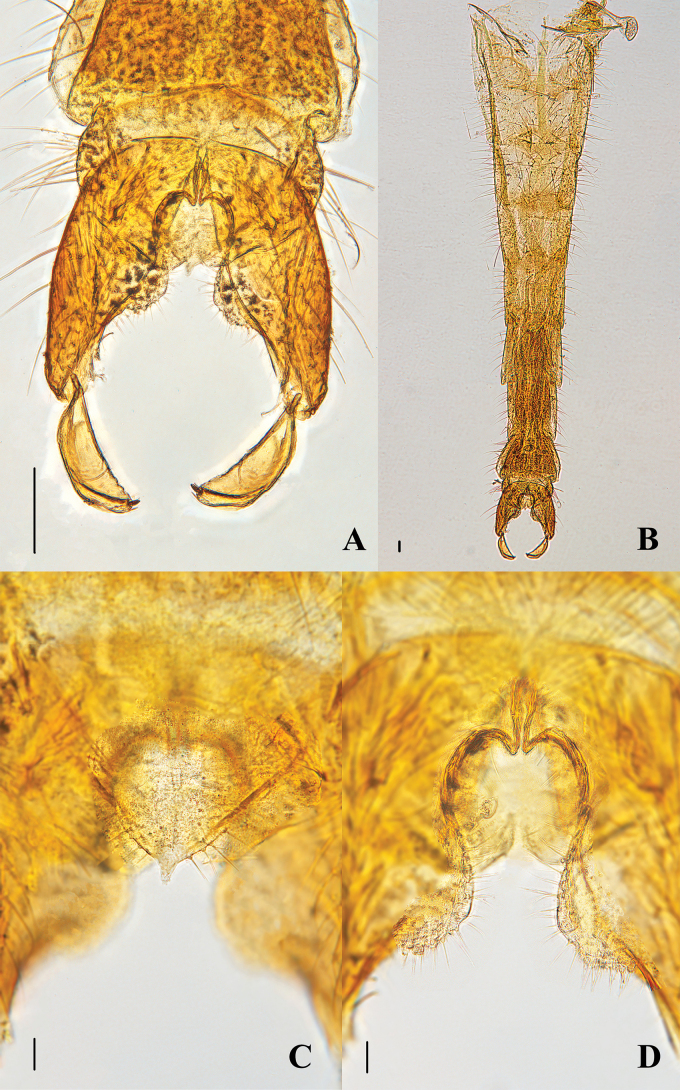
Psectrocladius (Psectrocladius) malum Liu, sp. nov., holotype male **A** hypopygium **B** abdomen **C** anal point **D** volsella. Scale bars: 100 μm (**A, B**); 20 μm (**C, D**).

**Figure 9.**
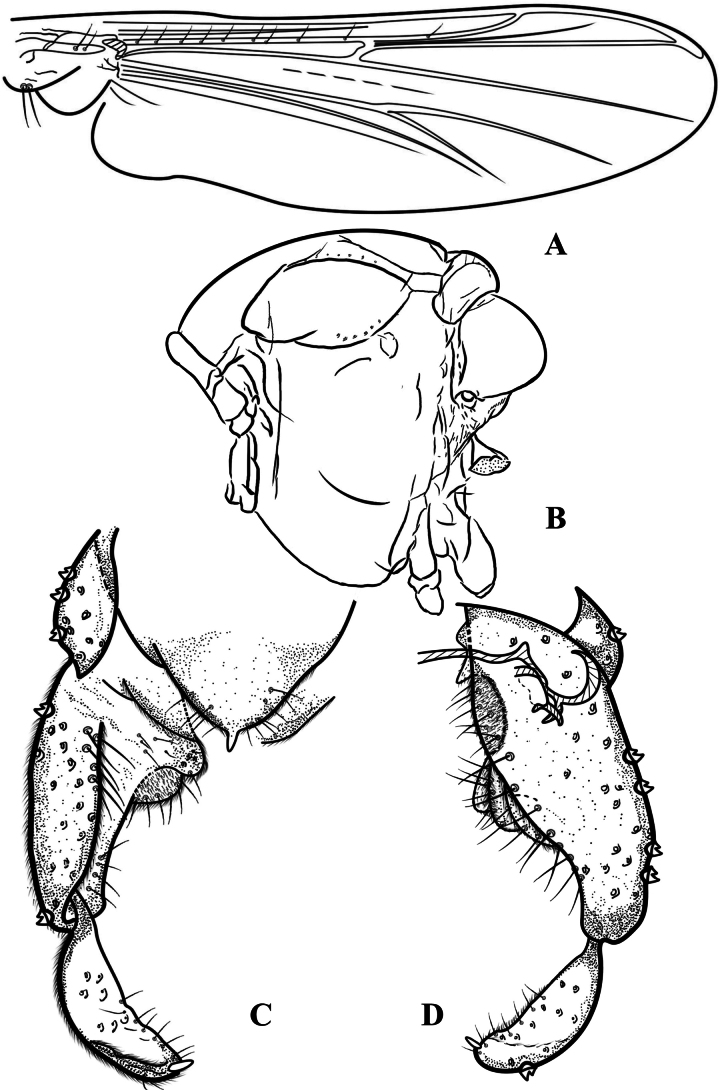
Psectrocladius (Psectrocladius) malum Liu, sp. nov., holotype male **A** wing **B** thorax **C** hypopygium, dorsal view **D** hypopygium, ventral view.

Hypopygium (Figs 8A, C, D, 9C, D). Tergite IX with 15 setae located two sides and the base of anal point. Tergite paratergital 11 setae. Anal point 14 µm long. The base of anal point 8 µm wide. Transverse sternapodeme 128 µm long, central slightly arched, with well-developed bilateral ossified processes, triangle-shaped. Virga 53 µm long. The end of inferior volsella pendulous and tongue-shaped. Outer margin of inferior volsella semicircular. Gonocoxite 303 µm, the base of gonostylus with a small convex, base outer margin of gonocoxite concave, the left and right contours form apple shaped. Gonostylus 166 µm, Megaseta 18 µm. HR 1.83; HV 2.45.

#### Distribution.

China (Inner Mongolia).

#### Etymology.

From the Latin, *malum*, apple, referring to the convex and concave upper inner margin of the two sides of the gonocoxite, which has an apple-like form.

#### Remarks.

The gonocoxite morphology provides crucial diagnostic characters for distinguishing *Psectrocladius* species. Psectrocladius (Psectrocladius) malum Liu, sp. nov. is characterized by a unique concave inner margin at the base of its gonocoxite, representing a distinctive apomorphic feature. This condition contrasts markedly with P. (P.) semicirculatus Sæther, 1969, which exhibits a more complex gonocoxite structure featuring both concave and convex inner margins. Furthermore, P. (P.) malum Liu, sp. nov. can be unequivocally differentiated from P. (P.) semicirculatus by its distinctive tergite XI morphology and anal point configuration.

Comparative analysis reveals that the anal point morphology of P. (P.) malum Liu, sp. nov. shows notable similarities with that of P. (P.) sokolovae Zelentzov & Makarchenko, 1988. However, these species can be readily distinguished by several quantitative characters: P. (P.) sokolovae possesses (1) a significantly higher antennal ratio (AR) and (2) a greater overall body length. Additionally, while P. (P.) barbimanus (Edwards, 1929) also displays a slight concavity on the outer margin of the gonocoxite base, this species is immediately recognizable by its distinctive pear-shaped overall morphology, providing a clear diagnostic feature for species identification.

### ﻿Key to males of *Psectrocladius* Kieffer from China

**Table:** 

1	Acrostichals present and distinct; tarsomere 5 dorsoventrally flattened	**2**
–	Acrostichals absent; tarsomere 5 laterally fattened	**4**
2	Shorter spur of mid tibia either about 2/3 as long as longest spur or lacking, anal point short to moderately long	**P. (Allopsectrocladius) obvius Wälker**
–	Shorter spur of mid tibia at most 1/3 as long as longest spur, anal point vestigial to short and strong	**3**
3	Degraded anal point, and AR > 2.0	**P. (Mesopsectrocladius) barbatipes Kieffer**
–	Anal point is short and rounded at the tip, AR 1.66–1.78	**P. (Mesopsectrocladius) wangi Liu, sp. nov.**
4	AR about 1.30, Mesonotum with traces of two shortened and darker lateral bands	***P* . (*Psectrocladius*) *formosae* Kieffer**
–	AR > 1.67, Mesonotum not as above	**5**
5	HR < 1.70, gonostylus curves inward from the end 1/3	**P. (Psectrocladius) longipennis Wang & Zheng**
–	HR > 1.70, gonostylus does not curve inward from the end 1/3	**6**
6	Abdomen yellowish brown, uniform color, tergiet XI left and right sides with reticulate pattern	**P. (Psectrocladius) gracilis Liu, sp. nov.**
–	Abdomen II–V yellow, abdomen VI–VIII brown, tergiet XI left and right sides without reticulate pattern	**P. (Psectrocladius) malum Liu, sp. nov.**

## Supplementary Material

XML Treatment for Psectrocladius (Mesopsectrocladius) wangi

XML Treatment for Psectrocladius (Psectrocladius) gracilis

XML Treatment for Psectrocladius (Psectrocladius) malum

## ﻿Additional information

### Data availability

All of the data that support the findings of this study are available in the main text.
